# A Newborn with Omphalocele and Umbilical Cord Cyst: An Interesting Entity

**Published:** 2014-08-15

**Authors:** Deepak Sharma, Srinivas Murki, Tejo Pratap

**Affiliations:** Department of Neonatology, Fernandez Hospital, Hyderabad, India

**Keywords:** Omphalocele, Umbilical Cord Cyst, Webbed Neck

**Fig. 1:**
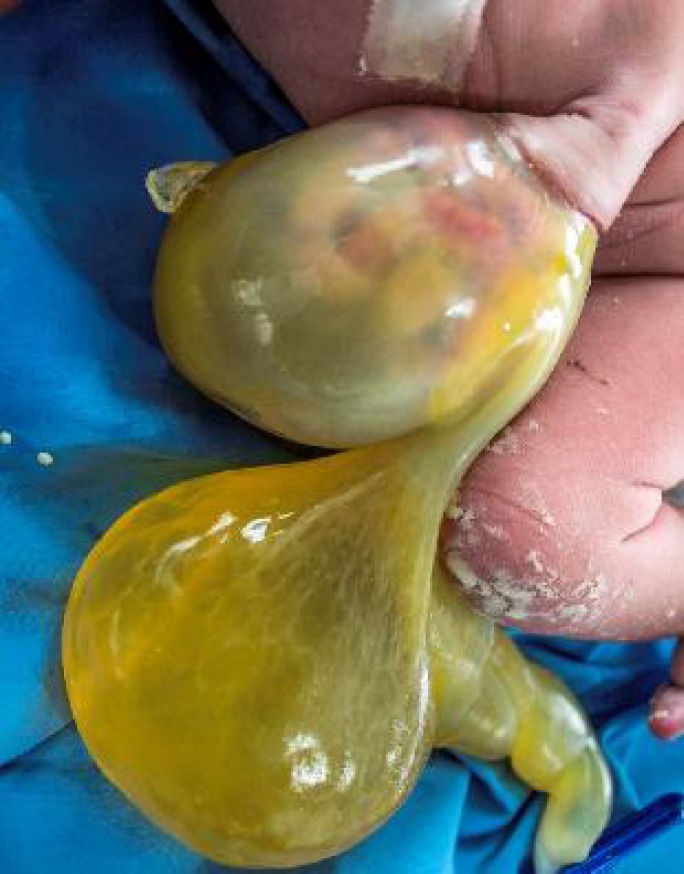
Omphalocele sac 14×10 cm with intestine as its content and umbilical cord cyst

**Fig. 2:**
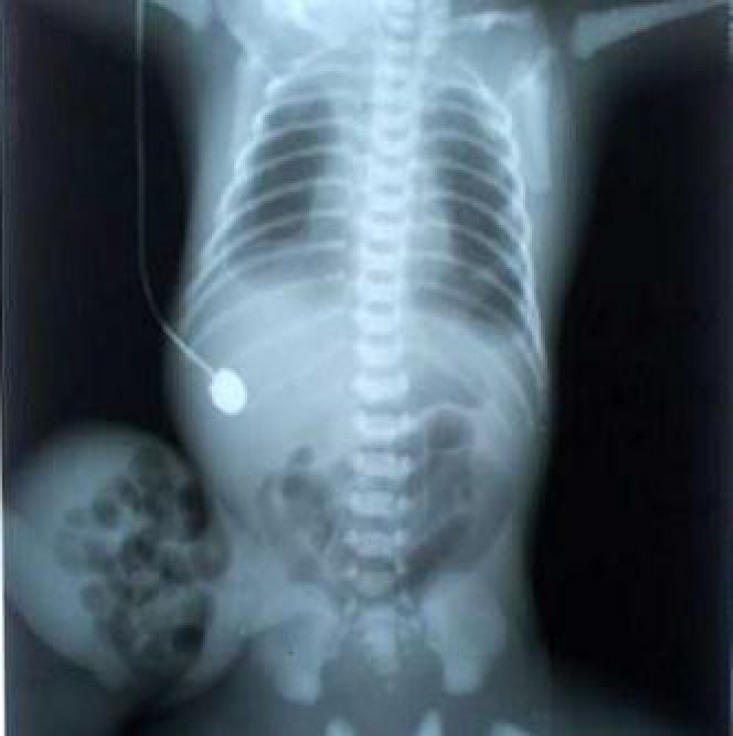
X-ray showing intestine as the content of the sac

**Fig. 3:**
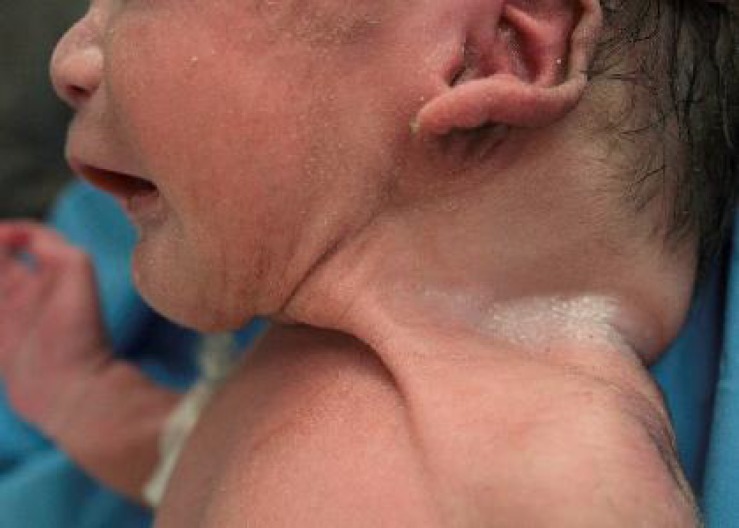
Short webbed neck of the infant

A late preterm male baby with a birth weight of 2.5 kg was born to primi mother. Baby cried immediately after birth with an Apgar score of 8/8/9. Baby was antenatally diagnosed as a case of omphalocele with umbilical cord cyst. There was no history of any drug intake or any other chronic illness in the mother. Baby was delivered by LSCS. On physical examination infant had an omphalocele sac measuring about 14×10 cm with intestine as its content and umbilical cord cyst (Fig 1). X-Ray showed intestine as content of the sac (Fig. 2). Transillumination test was positive. Baby had webbing of neck (Fig. 3) and no other malformations.

Infant was further evaluated with ECHO and Neurosonogram which were normal. Baby was operated on day 2 of life and intestines were reduced. Baby was gradually started on feeding and post-operative course was uneventful. Karyotypic analysis of the infant was normal. The infant was discharged successfully and is now in follow up.

 Omphalocele also known as exomphalos is a midline defect characterized by the evisceration of abdominal contents covered by a protective sac. The wall of the sac is formed by (inside to outside) peritoneum, Wharton’s jelly and amnion^[^^1^^]^. The defect is most commonly located at the base of the umbilical stalk in the midline. In omphalocele, the bowel does not return to the abdominal cavity between 10-12th week of gestation^[^^2^^]^. 

The incidence of omphalocele ranges from 1.5 and 3 per 10,000 births^[^^3^^,^^4^^]^. Associated anomalies in omphalocele range from 50% -70% and are most important determinants in prognosis^[^^5^^]^. 

 The treatment involves stabilization and surgical reduction of the sac content.

 The syndromes associated with omphalocele include^[^^6^^]^:

• Otopalatodigital syndrome type II,

• Melnick-needles syndrome, 

• Rieger syndrome,

• Meckel syndrome,

• Shprintzen-Goldberg omphalocele syndrome,

• Lethal omphalocele-cleft palate syndrome, 

• Cerebro-costo-mandibular syndrome,

• Fetal valproate syndrome, 

• Marshall-Smith syndrome, 

• Fryns syndrome, 

•Donnai-barrow syndrome, 

• Charge syndrome, 

• Goltz syndrome, 

• Carpenter syndrome, 

• Toriello-Carey syndrome, 

• Cornelia de Lange syndrome,

• Sprengel anomaly,

• Kennerknecht syndrome.

***What should be done?***

• Perinatal identification of omphalocele should be evaluated for omphalocele-related disorders and familial inheritance and a thorough genetic counseling should be done promptly for the parents.

• Omphalocele associated malformations should be searched as there are very high chances of having them and help in prognostication of the parents.

• Karyotypic study should be done to rule out chromosomal disorders associated with omphalocele. 
